# Object Detection Techniques Applied on Mobile Robot Semantic Navigation

**DOI:** 10.3390/s140406734

**Published:** 2014-04-11

**Authors:** Carlos Astua, Ramon Barber, Jonathan Crespo, Alberto Jardon

**Affiliations:** System Engineering and Automation Department, University Carlos III, Av de la Universidad, 30, Madrid 28911, Spain; E-Mails: pato15cr@yahoo.com (C.A.); jocrespo@ing.uc3m.es (J.C.); ajardon@ing.uc3m.es (A.J.)

**Keywords:** mobile robots, semantic navigation, object detection, contour detection, descriptor detection, image correlation

## Abstract

The future of robotics predicts that robots will integrate themselves more every day with human beings and their environments. To achieve this integration, robots need to acquire information about the environment and its objects. There is a big need for algorithms to provide robots with these sort of skills, from the location where objects are needed to accomplish a task up to where these objects are considered as information about the environment. This paper presents a way to provide mobile robots with the ability-skill to detect objets for semantic navigation. This paper aims to use current trends in robotics and at the same time, that can be exported to other platforms. Two methods to detect objects are proposed, contour detection and a descriptor based technique, and both of them are combined to overcome their respective limitations. Finally, the code is tested on a real robot, to prove its accuracy and efficiency.

## 1. Introduction

There are many research projects considering the problem of recognizing different objects [1–4]. Most of these projects work under certain conditions, where there is a finite number of objects and the environment is somewhat controlled [5]. This is because it is extremely difficult to program a robot to recognize any object that it could encounter and on the other hand, creating a database with thousands of objects is not always the best solution. Another feature to take into account, is the fact that the lightning conditions of the environment can greatly affect the object recognition ability [6], thus making the task at hand even more challenging.

**Figure 1.**
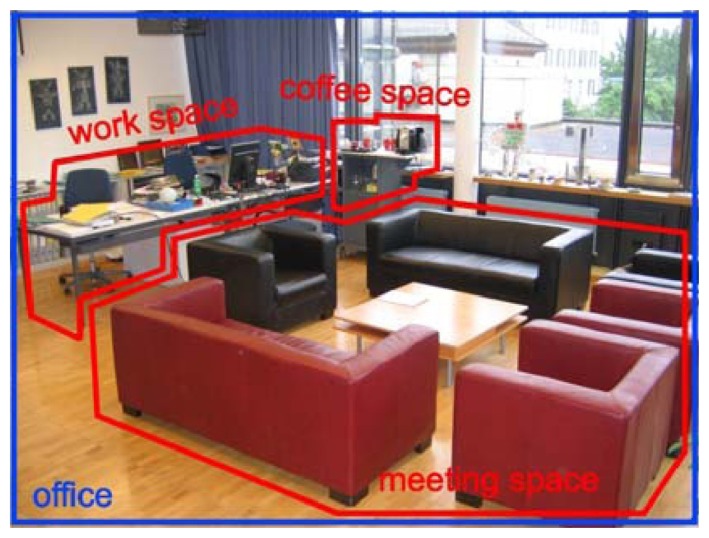
Differentiate between objects in environment [11].

Since every project tries to do object recognition in its own way, each project has to decide which is the best approach to achieve its final goal. The current state of technology provides different ways to achieve this task however, there are no projects that can provide these days, a somewhat intelligent algorithm to recognize objects based only on vision. It has to be noted that objects for humans have a meaning because they have interacted with them previously or because they can generalize what a new object could be, based on previous experiences. Most humans have encountered a situation where a new object is presented and the meaning of this new object can only be created in their mind after an interaction has been made or an explanation of the object is given. Interacting with objects is not a part of this work however, since this is applied for mobile robots, the fact that the robot can move creates a different scenario where this motion can be used to enhance the task. The problem then changes from merely detecting objects to how to apply this to a mobile robot. Also, the need to detect objects is because the mobile robot would be used for semantic navigation [7] and detecting objects to create maps [8] is one of its key factors. The application of the proposed algorithm is to allow a mobile robot to differentiate between the objects in a scenario to obtain properties, which would be used to assign them a meaning and use this information for semantic navigation [9]. The main difference between this approach compared to manipulation robots, is that the requirements for a detection mechanism in mobile robots are lower, because the amount of information needed is not as high [10]. As it is shown in Figure 1, the critical part for this application is to assign a meaning to the different parts of a room, not to accurately detect the size of an object.

As mentioned before, object recognition is very important in robotics at the present time and in the case of this paper, applying it to mobile robots generates a somewhat unique scenario, where the algorithm has to provide some features that may not be needed if the type of robot changes. Since most mobile robots do not have heavy computer power and the response time to detect an object should be sufficiently efficient, so the robot can move fairly quick, then the generated code has to try to work under these specifications. The code was generated using computer vision algorithms that are not heavy and at the same time, providing an adequate response time. The code then is applied on a real robot and two experiments are carried out so it can be determined if it works properly.

## 2. Object Recognition

Object recognition [12] is widely used in machine vision industry for inspection, registration and manipulation tasks. In many industries [13], robot arms need a mechanism to recognize objects to act on them, in an autonomous way, but the algorithms for object recognition have many limitations because of the changes in illumination, occlusion, scales and positions. Because of these limitations, there are many research groups working on projects to provide efficient solutions for object recognition in many fields [14–17], and robotics is the one where this project will be focused. Since object recognition will be used on a robot, choosing an adequate software and hardware platform is a key factor. As many projects are being developed at universities, open-source software and cheap hardware has become a trend because it lowers the costs [18,19]. Based on this fact, using trends when deciding on software and hardware gives a good idea what most researchers agree is better, where most development is happening and where more literature and options are available. This is why, the Kinect is used for image capture [20], the Robot Operating System (ROS) [21] was selected as the operating system, C++ is the language used as it can also be exported to other environments and The Open Source Computer Vision (OpenCV) [22] handles image processing.

### 2.1. Contour Extraction Concept

Contours are the shapes of different objects, humans use this type of data to determine what a given item is [23] and it is just one way of how humans discriminate what they are seeing. Applying the same concept to robots and since the Kinect is the camera used, the best type of image to accomplish this task is the depth image.

**Figure 2.**
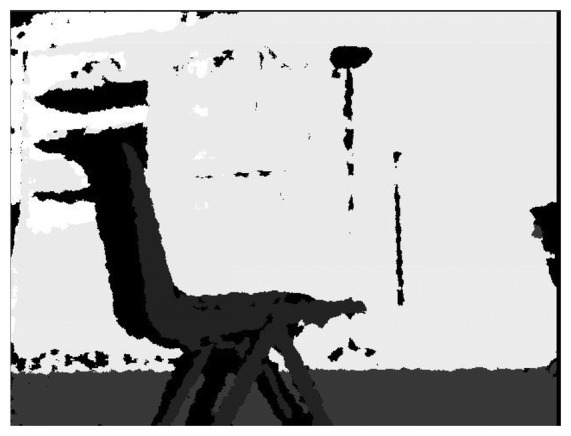
Depth image provided by the Kinect.

The depth image provided by the Kinect, like the one shown in Figure 2, is a combination of the infrared sensor and RGB camera and it shows all the pixels of the image with a gray value that indicates how far away each pixel is. Once a contour is extracted, OpenCV is used as it provides different types of comparison that can be used, like the area, the signature, neural networks and correlation, amongst others. Considering that this work would be used for semantic navigation, there should be a way to generalize object detection as it would be very difficult to include all the shapes a mobile robot can find in any given environment. From these methods, the one that provides better features is correlation, since the area can get objects mixed up with similar area values, the signature is very specific and neural networks require to be re-trained every time a new object is added. As image processing is basically handling digital images as if they were matrices, an image would then be a matrix with certain amount of rows and columns and each of the cells would contain a value, showing the level of the specific pixel. The values contained in the matrices are the ones used for the correlation, where it would compare the value of the pixels of the model with the value of the pixels of the image and it would provide a correlation matrix, which shows how similar a given matrix is compared to the image.

### 2.2. Descriptor Concept

Image processing is a very difficult task since scenarios are constantly changing. This is not referred to changes like adding new objects or where things are moved towards, but even if a picture is taken from the exact same scenario, there are differences caused by factors like illumination or noise. Because of this problem, the task of finding similarities between two images of the same scene or object has become a major problem in computer vision applications. When it comes to image matching, extracting information that can be used to accurately match different viewpoints of the same image is necessary. Obtaining certain characteristics that can be used to match these objects or scenarios is called feature detection [24]. Feature detection occurs within an image and tries to describe only those parts of the image where unique data or signatures can be obtained (descriptors). To do this, first “keypoints” are extracted from different-unique locations of the image like borders, blobs, *etc.* and these points should be highly repeatable. Then, neighborhood regions are chosen around every keypoint represented by a feature vector and unique feature descriptors are computed from each region. These descriptors have to be different/unique and at the same time robust to noise, to detection displacements and to geometric and photometric deformations. A different set of feature detection algorithms has been proposed to compute reliable descriptors for image matching and within them, the Speeded Up Robust Features software (SURF) [25] is one of the mostly used nowadays. The matching of these descriptors is based on a distance between the vectors and it is the main factor to consider when figuring out if the matches found are accurate. To do this, the Fast Library for Approximate Nearest Neighbors (FLANN) [26] is an example of a library used to perform fast approximate nearest neighbor searches.

### 2.3. Current ROS Object Detection Algorithms

There are many applications developed for object detection with ROS and there are other research projects currently in progress, like RoboEarth [27], Tabletop Object Detector Project, Cob Object Detection Project, and Rail Cv Project, among others. They use point clouds as their core mechanism. Then, they apply different methods to discriminate the objects present in the point cloud in order to use this information to accomplish different tasks. There are other packages that try to use different image data to locate objects, for example: Objects of Daily Use Finder. This package uses descriptors to define if an object is present in an image and it can create a square around the object for recognition. Most of the applications and packages developed in ROS are developed for robotic manipulation, which require high precision images, leading to the necessity of high computer power. In the case of mobile robots, high computer power is not always attainable because these robots have to be small, so they can move in most places and to keep an efficient power supply autonomy. Also, the generalization needed for a mobile robot can be reached with point clouds, since they generalize using the shape of an object, the problem is that the amount of time needed to do this, while the robot moves, is forbidden.

## 3. Implemented Techniques

Both methods were developed and then analyzed to find what they had to offer and of what they lacked of. The way the algorithms were implemented is explained below using pseudo-code. Then, both methods are compared.

### 3.1. Implemented Contour Extraction Algorithm

First, NodeHandle has to be initialized. Afterwards, an OpenCV display window has to be created to handle the images. The next step is to create an ImageTransport instance that would be initialized with NodeHandle. This is done because images cannot be imported directly in C++ code. Then it subscribes to the topic where the image is published.

**Figure 3.**
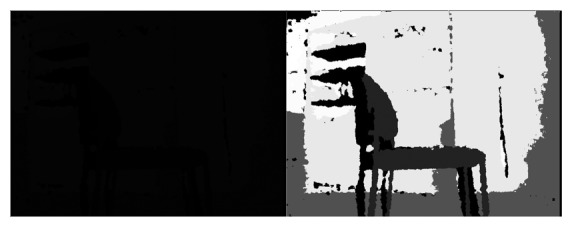
Image obtained from the kinect(left hand side) compared with equalized image (right hand side).

The callback (pseudocode below) would only have to manage a type called sensor_msgs/image, that controls all the image encoding and decoding. The image is hold until the program unsubscribes from it. Once the callback function is called, the ROS image is converted to OpenCV with a pixel encoding. Since the gray tones were difficult to discriminate, the image was equalized. This is because the black image in Figure 3 was actually the one provided by the Kinect and the one on the right is the result after equalizing it.

*openCV formatimage* = *convertOpenCV* (*image_received*)*image* = *convert2OpenCV* (*BLACK_WHITE_CODE, image*)*image* = *openCV Equalize*(*image*)*Mat Format image_2* = *convertMatFormat*(*image*)*Erode*(*image_2*)*Dilate*(*image_2*)**for**
*pixel_i*
**do** //This if for all pixels in the image **if** (*isTooFar*(*pixel_i_*)) **then**  *pixel_i* ← 0 **else**  *pixel_i* ← 1 **end if****end for***contour_selected* ← *getBiggerContour*(*arrayContours*)*get BoundigBox*(*contour_selected*)*correlate*(*image_2, models, results*)*Point extrema* = *getExtrema*(*results*)**if** ((*extrema.x > extrema.y*)*OR*(*extrema.x > extrema.z*)*OR*(*rangeFullFills*())) **then**
*print*(“*ObjectDetected*!”)**end if**

**Figure 4.**
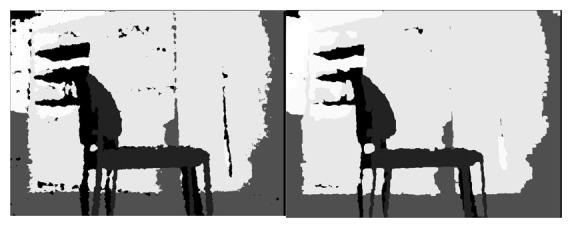
Morphological transformations comparison.

The image is then converted to a Mat type, then OpenCV is used to treat it. Then morphological transformations are implemented to enhance the information needed, as shown in Figure 4.

**Figure 5.**
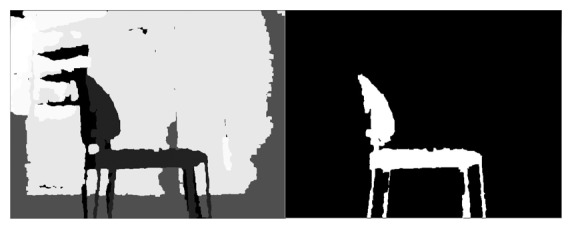
Comparison with binarized image.

The next step is to extract the closer objects based on the gray value, which shows the distance. The result is a binary image where 1 (white) shows the important pixels and 0 (black) shows the rest of the pixels. As the robot can move, detected object can be limited to only the ones within certain distance, as shown in Figure 5.

**Figure 6.**
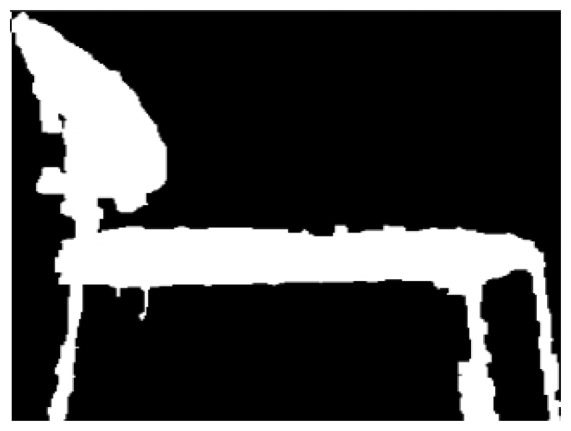
Biggest contour extracted.

At this point, the code calculates which pixels are connected together and it extracts the biggest contour. This is done by calculating which contour is the biggest, then masking out the specific region and extracting this mask to another image. This can be seen in Figure 6.

Once there is an image with only the biggest contour, correlation is applied to the models. The correlation matrix gets its extrema extracted and the extrema values of all the models are compared between them and a range. If the result is valid, it shows a message than an object has been detected.

### 3.2. Implemented Descriptor Algorithm

The second part of the paper uses descriptors to detect objects (pseudocode below). The algorithm follows the same steps as the Contour Extraction Algorithm up to where the image is converted to OpenCV format. The only difference is the topic where the code subscribes, which now is the /camera/rgb/image_mono, since descriptors do not need color. Once the image is stored, then keypoints are extracted from it and all the models. These keypoints are used to find descriptors on both the image from the camera and the models, which would be compared using FLANN.

*key Point s* = *KPextraction*(*image_received*)*matches*[] = *FLANN_Match_Model*(*image_received*)*sum* = 0**for**
*i* = 0; *i* < *matches.size*(); *i* + +) **do** **if** (*distance(matches*[*i*]) < *DISTANCE_PREFIX*) **then**
*sum*+ = *distance*(*matches*[*i*]) **end if****end for****if** (*sum* > *EMPIRICAL_DATA*) **then**
*print*(“*ObjectDetected*!”)**end if**

After matches have been found, the distances between the matches and its amount are used to calculate if the object is present.

**Figure 7.**
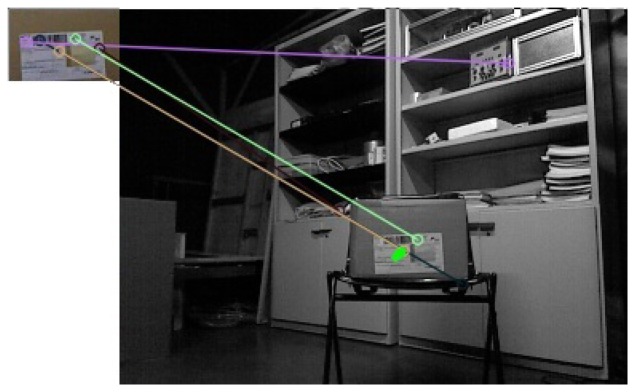
Model descriptors matching an image.

These values are used since the algorithm is able to find matches that do not correspond to the object, as shown in Figure 7.

### 3.3. Contours vs. Descriptors

Contours are very well suited where a shape clearly represents an object and they allow a certain level of tolerance, since a value of how many objects have to be similar can be added. Also, computationally it is not heavy, allowing a fast response. Both of these features are valuable in mobile robots, since it improves its power supply autonomy and it decreases weight, because a smaller computer can be used. Another advantage is using the depth image from the Kinect, because light changes do not affect the method used. The problem with this algorithm arises when a shape does not discriminate an object. Descriptors in fact, provide the ability to detect specific objects, regardless of light conditions, scale or rotation. The problem is that they do not allow to generalize, which is needed for semantic navigation.

## 4. Combination of Techniques

A different feature to take into account when combining techniques is the ability to add new objects in an efficient way. In case new objects had to be added to the algorithm, the code would need minor changes and it would be simpler than having to train again a neural network for example. Furthermore, the same object can be added on both algorithms so it can detect it with both of them, in case the robot needed such skill. The skill to use two different mechanisms to detect objects is what makes this paper different from the options available for ROS. This does not mean the options available are not considerably very well designed, it just means that for the specific application this work covers, the approach proposed would be a better solution in cases where similar restrictions are present.

Previously the current options available for object detection with ROS were mentioned. The options provide good results but were not adapted to mobile robots. The algorithm proposed for this paper takes advantage of some of the object detection algorithms available and it uses them to provide a more suitable solution for mobile robots, especially for those cases in which these robots do not have a lot of processing power or where the available algorithms do not have some sort of generalization. This is the main difference between this work and other solutions available.

## 4.1. Algorithms to Combine Techniques

**Figure 8.**
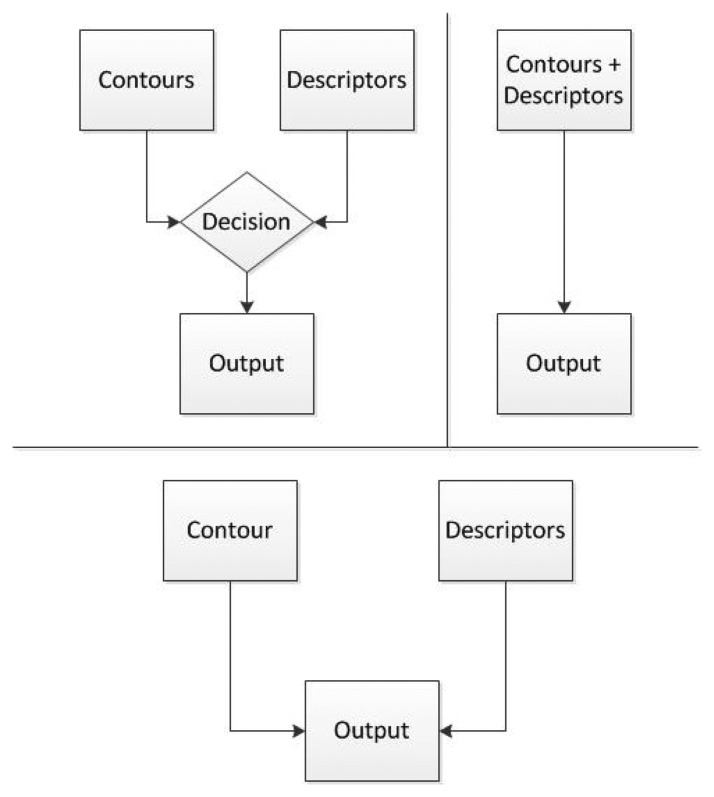
Methods to combine techniques.

There are different ways to combine both methods to provide better results than each one separately. In this paper the methods that have been considered are described above and a diagram describing them is shown in Figure 8.

1. **Switching:** Switching refers to the ability to change from one method to another when needed. This approach would use a condition that would choose which one of the methods is more appropriate for the current object. The downside is the fact that there should be a way to tell which of the methods is going to be better, every time a new object is detected. This decision is not simple to program and it incurs in a bigger complexity of the program.
2. **Merger:** Merge implies that both algorithms would be used to detect the object. This approach would create a common area for both methods to be able to decide whether the object is the right one or not. The method can be applied by using data coming from contours and detectors and for the decision taking task it will be using the results provided by both. For example, if the contour of an object is rectangular and its descriptors show a box, it would probably be a box but if the contours are triangular, then it will probably not be a box. The disadvantage of these method is the ability to generalize from the contours, given that for every contour there should also be a descriptor image and descriptors are very specific.
3. **Accuracy:** Create a method to define which of the two algorithms proves to be better for each object. This is, in fact, the method implemented in this work because each object can be added to one or another, depending on the results needed. This is useful because an object can be better recognized using contours than descriptors or vice versa, and the user can choose which one works better for its application.

### 4.2. Proposed Algorithm

**Figure 9.**
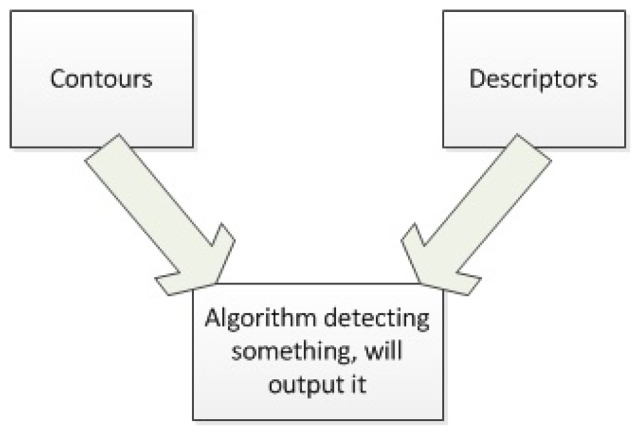
Merging of techniques.

From these options, merger was selected as shown in Figure 9 because it provides the possibility to add objects to only the portion of the code that will detect them better and it gives the opportunity to add the same object to both algorithms in case this is needed. Switching would create the problem to add some sort of condition to calculate which of the algorithms is better for each case and accuracy would have to figure out somehow which is providing better results while running.

## 5. Experimental Results

**Figure 10.**
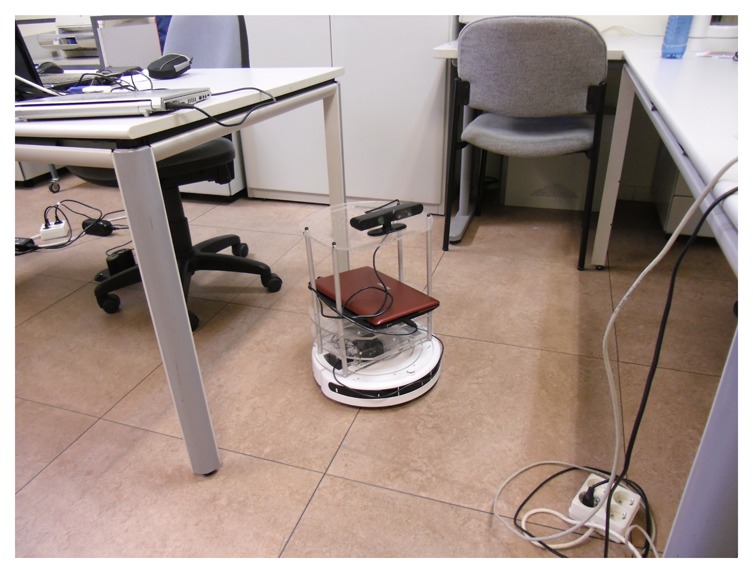
Turtlebot.

The platform where the algorithm is going to be implemented is a robot called Turtlebot, which is made up of a Roomba base, a Kinect attached to it and a laptop that handles all the communications between the devices and where Linux and ROS are already installed. An image of the Turtlebot is presented in Figure 10. The idea is that the robot can move around and detect objects while it is moving. The objects should be located in a position where the Kinect can see them properly and all the tests are going to be carried out with this robot.

### 5.1. Semantic Scenarios

The semantic scenario used to test the efficiency of this project was a laboratory. The laboratory provides a wide range of objects that can be found in it, such as: chairs, tables, computers, tools, among others and therefore, different experiments can be tested. Also, the scenario is related to an experiment presented in the semantic navigation paper [28] where this object detection code is applied. This would allow a robot using the semantic navigation algorithm described in [28] to explore and perform a recognition task, allowing it to detect the place where it is located and where it should go. Furthermore, the objects selected for the two experiments were chosen according to objects that can be found on different laboratories.

**Figure 11.**
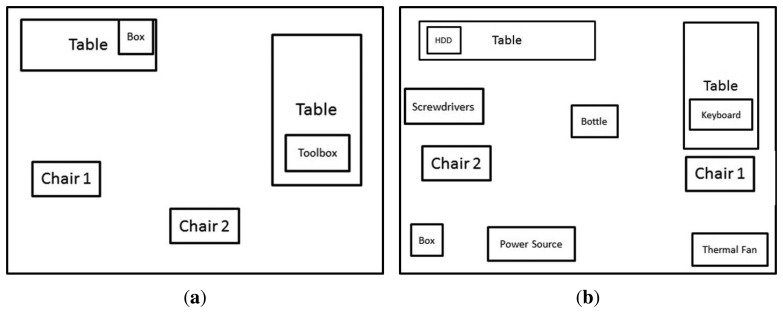
Scenarios 1 and 2. (**a**) Scenario 1; and (**b**) Scenario 2.

The first experiment was designed to contain only a few objects as shown in Figure 11a. The scenario was just created to test both algorithms working together. The second scenario, shown in Figure 11b, was developed to expand the capabilities of the first one and to calculate whether this approach could be applied to larger groups of objects.

### 5.2. Components

**Figure 12.**
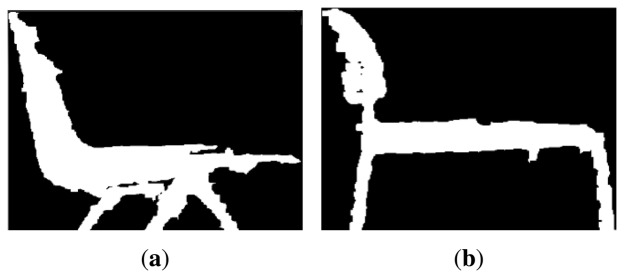
Experiment 1, Chair. (**a**) Experiment 1, Chair 1; and (**b**) Experiment 1, Chair 2.

**Figure 13.**
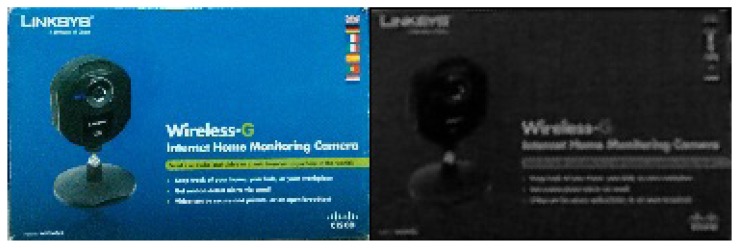
Experiment 1, Box.

**Figure 14.**
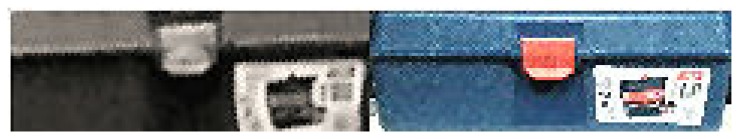
Experiment 1, Toolbox.

The first scenario where the test is run, Figure 11a, was designed with only a few objects to test its efficiency. It was created so it could detect two different chairs, a toolbox and a box. All the objects were chosen randomly from what was available at the laboratory. The objects selected are shown in Figures 12, 13 and 14.

Figure 12a represents one of the chairs located at the laboratory. The image was taken with the Kinect and is the biggest contour extracted. The reason why the edges are rough is because the camera has problems detecting curve contours, but even so, the chair can be clearly distinguished.

Figure 12b represents a different type of chair, as it has a different contour. The code should be able to tell one chair from the other based on this.

Two tests were run considering a box (Figure 13) and a toolbox (Figure 14). They were chosen randomly and the reason why it is used in the descriptor portion of the algorithm is because a rectangle does not clearly tell if an object is a given object.

All these objects are the models inserted in the algorithm and they look different because the type of images depend on the part of the algorithm were they were inserted. In the case of the chairs the algorithm takes these two images and compares them with the biggest contour extracted from the Kinect. If the result of the correlation is satisfactory, then the program would display a message that one of the chairs has been detected.

**Figure 15.**
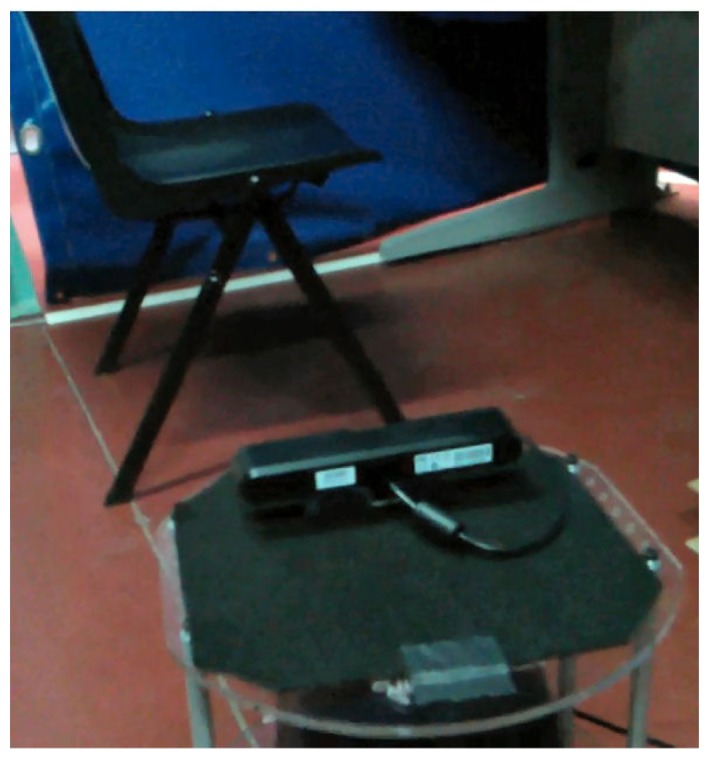
Moving robot and detecting chair.

An image of the robot detecting the chair while moving is shown in Figure 15.

As for the box and the toolbox images, these are images taken with the Kinect from the objects detected with descriptors. Since descriptors use more specific information from the models, the images show more details about the objects.

The second scenario, Figure 11b, was created so it can be tested with more objects which would tell whether this would work for larger amount of objects or not. For this experiment more objects were randomly included to those of scenario 1.

**Figure 16.**
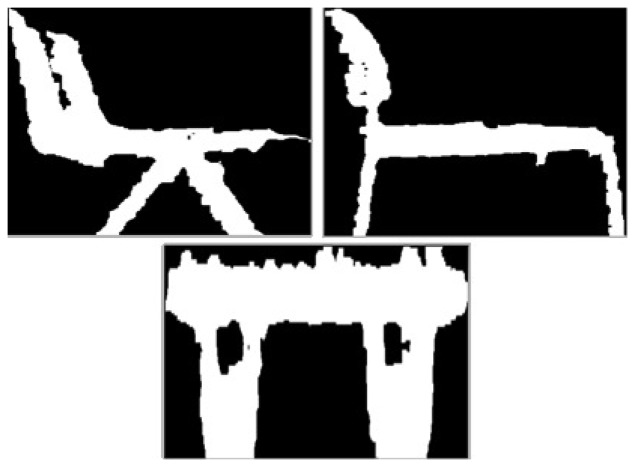
Experiment 2, contours selected.

The two images at the top of Figure 16 are the two chairs used on the first experiment. A table was added. It can be noted that the contours can be differentiated from one another.The images are also taken from the extracted contour given by the Kinect. Even though, the edges are irregular, this is how the Kinect sees the object.

**Figure 17.**
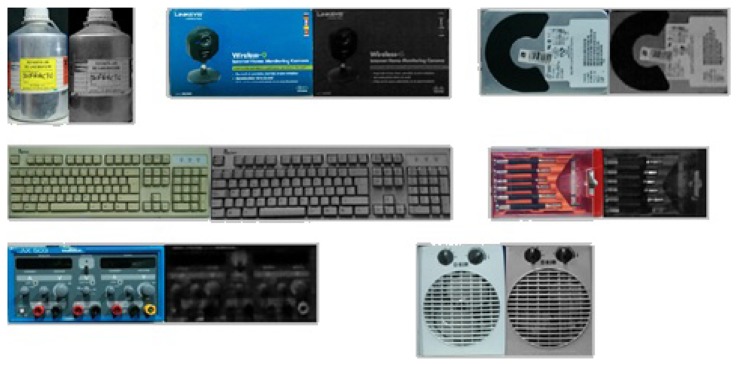
Experiment 2, objects selected.

**Figure 18.**
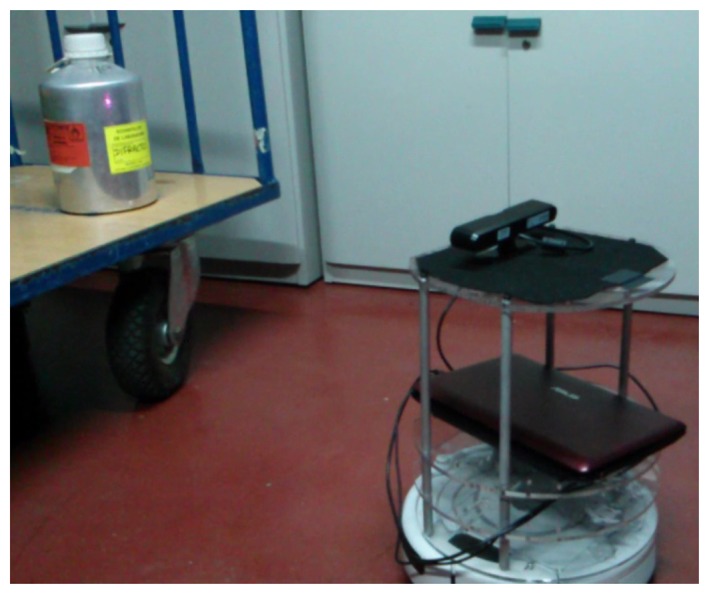
Moving robot and detecting bottle.

The objects used for descriptor detection where: bottle, box, hard disk drive (HDD), keyboard, screwdriver, power source and thermal fan, as shown in Figure 17. There are two images for each object, one is the real object (right hand side) and the other one is the image obtained from the kinect (left hand side). An image of the robot while detecting the object is shown in Figure 18.

### 5.3. Results of the Experiments

**Figure 19.**
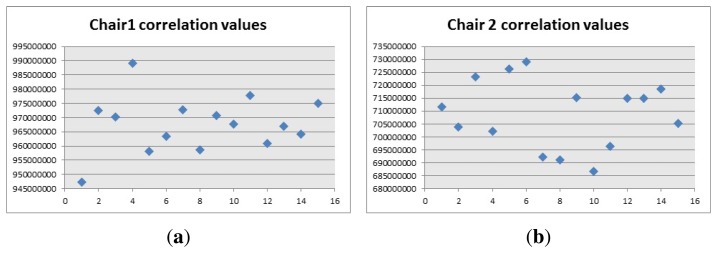
Correlation values. (**a**) Correlation values, chair 1; and (**b**) Correlation values, chair 2.

For the first experiment, the values of correlation for both types of chair were obtained (Figure 19).These could be used to tune the algorithm into separating objects Angela and belong to Figure 12 respectively.

These values have no units and the results give really high values because when comparing images of the same type,black and white images, the matching pixels have the exact same color as the original. The values obtained are those when the object is the biggest contour of the image. Therefore, they are only applied when a chair is being correctly captured with the Kinect.

As shown in Figure 19, the correlation values for chair 1 are above 945 × 10^6^ and the values, and for chair 2 are above 685 × 10^6^. These values were chosen based on empirical data, which was obtained comparing the data when the contour was the correct one, with other contours and these can be used in the algorithm to create a condition where the correlation of the biggest contour has to overcome a certain value, to be considered as a positive match. These values can also be used to generalize in certain scenarios where other objects give a lower value with the same models, providing the possibility to get matches from other objects even if they do not look much alike the model.

Once the proper values were included in the algorithm, the chairs were properly detected and they could be differentiated between them. The only problem appeared when two objects were detected as the biggest contour, because the results were inaccurate. For example, if a person was touching the chairs, the person was considered part of the object.

**Figure 20.**
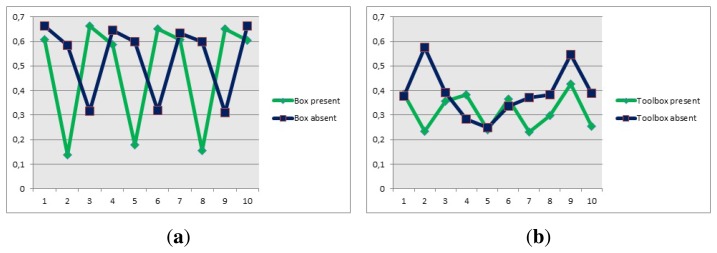
Descriptors distance. (**a**) Box descriptors distance; and (**b**) Toolbox descriptors distance.

From the descriptors point of view, the descriptors of the models represented in Figures 13 and 14, for the box and the toolbox match pixels in the image obtained from the Kinect. Even though these matches are not real matches, the distance was the key factor to calculate whether the object was present in the image or not. The distances obtained from all the descriptors is showed in Figure 20, when the objects are present or absent in the image.

The distance obtained is useful since the descriptors are closer when the object is present in the image. This distance is used as the discrimination value and it was included in the algorithm, to prevent false positives. In this case, the fact that a person holds the item or if it is located on a table, did not prevent the object from being detected properly. The only problem is in the case of the toolbox, because the distance when the object is present and when it is absent are very close to each other. This caused that the value chosen to be discriminated was very hard to reach, thus detecting the toolbox was a little harder than the other objects and the robot had to position itself closer to the object to detect it properly

For the second experiment, the correlation values for the two chairs and the table, shown in Figure 16, were obtained when the object was in front of the camera and it was the biggest contour.

**Figure 21.**
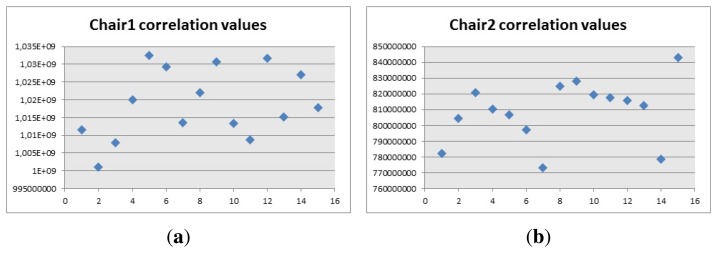
Correlation values, experiment 2. (**a**) Correlation values, chair 1; (**b**) Correlation values, chair 2.

**Figure 22.**
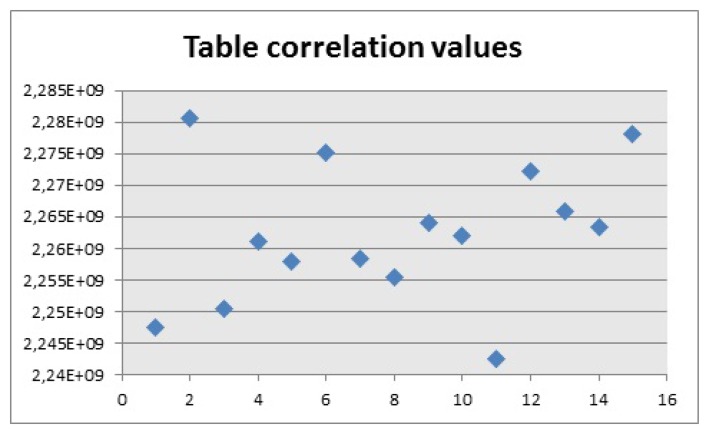
Correlation values, table.

For the first chair, Figure 21a, the values of the correlation are around 1 × 10^9^, which is why the correlation algorithm used a range between 1 × 10^9^ and 10 × 10^9^ as the values to calculate if there is a correct detection. The reason why the range uses a low and high value is because if there are objects that cover most of the pixels represented by the chair, the algorithm can detect them as a positive match. By applying this range, the algorithm makes sure that the results are accurate and the contour extracted is effectively similar to the model. In the case of the second chair, Figure 21b, the values are around 770 × 10^6^ and the values for the table, Figure 22, are close to 2.2 × 10^9^. Then the values chosen for the ranges are 700 × 10^6^ and 850 × 10^6^, for the second chair and 2.2 × 10^9^ and 2.4 × 10^9^ for the table. It should be noted that the extraction of the contour has to be carried out so it looks like the model and the experiment showed that looking at the objects from an angle, sometimes provided distorted images that did not match the model.

The descriptors portion of the algorithm turned out to be more challenging. The reason is because SURF and FLANN are able to find good matches even if the object is not present. Also adding more objects to the algorithm made the task of object detection incur in many errors and it would detect objects where there are none. Because of these errors, the discrimination part of the algorithm was modified so it could accurately detect the objects. The modification carried out was to use the amount of descriptors extracted from the model and compare the distance of the descriptors when the object was in the image and when nothing was there. The task of calculating the amount of descriptors and distance between them had to be carried out for each model individually, since one object had 316 descriptors and others only had 4. The distance between the descriptors for each object was then used to calculate how many of them should be close to each other to calculate if the object was in the image.

**Figure 23.**
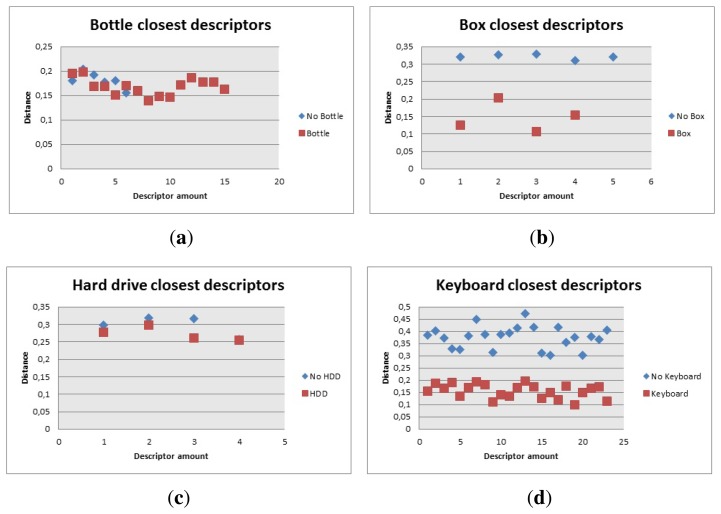
Closest descriptors with and without object. (**a**) Closest descriptors with and without bottle; (**b**) Closest descriptors with and without box; (**c**) Closest descriptors with and without hard drive; and (d) Closest descriptors with and without keyboard.

As shown in Figure 23a, the value of the closest descriptors cannot be used to tell wether the object is present or not, however there are more descriptors close to each other when the object is in the image. The algorithm was then modified to count how many of those descriptors should be in the image to consider if bottle, Figure 18, was present or not. Having in mind that the bottle had 89 descriptors, it was determined that there should be more than 15 close descriptors to tell if the bottle is present.

The box had 17 descriptors and the amount was not as critical as the distance between them, as can be seen in Figure 23b, so the algorithm had a maximum distance of 0.17 to be considered a close descriptor and there should be three or more to be considered a good match.

The keyboard, Figure 23d, provided 316 descriptors. The distance of the closest descriptors was considered for the first 100. This information was used as a reference to calculate how many descriptors had to be used to tell if there was a good match. The amount of close descriptors used was 73 and they had to have a distance lower than 0.2.

The hard drive, Figure 23c, gave 27 descriptors and the code was modified so the distance for the descriptors to be counted was lower than 0.26 and there should be more than four of them, As a matter of fact, the original code was modified because the algorithm failed to recognize the hard drive properly and gave wrong results when other objects were present in the image, so the amount of descriptors needed for a positive match increased, which then caused that the robot needed to be closer to the HDD to recognize it properly.

**Figure 24.**
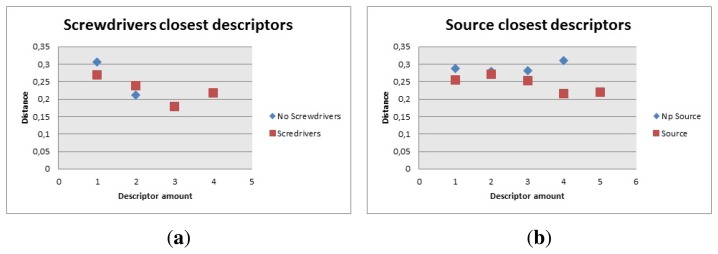
Closest descriptors with and without object (screwdrivers and power source). (**a**) Closest descriptors with and without screwdrivers; and (**b**) Closest descriptors with and without power source.

The screwdrivers, Figure 24a, had 29 descriptors but in this case, it was a little difficult to calculate how many to use because only four of them gave low distance values. The algorithm then was modified to count the ones under 0.24 and there should be more than two to consider it a good match.

For the power source, Figure 24b, the amount of descriptors was 17. The distance was set to be less than 0.25, and the amount of descriptors detected under this distance had to be more than four.

**Figure 25.**
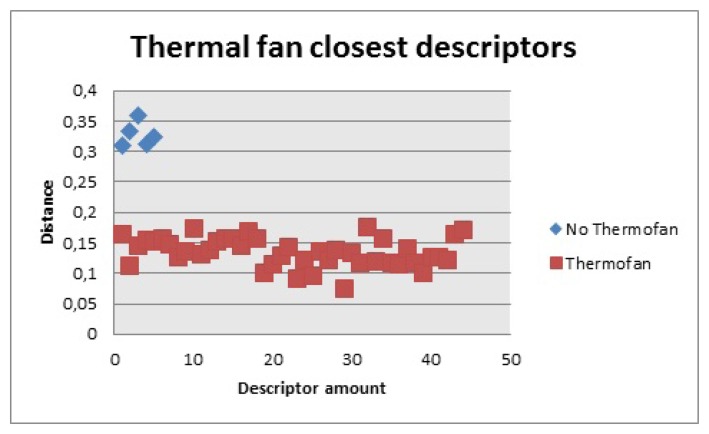
Closest descriptors with and without thermal fan.

The thermal fan, Figure 25, had 314 descriptors The closest ones for the first 100 were considered. Based on this fact, the distance for the closest ones had to be less than 0.18 to be counted and there had to be more than 30 to be considered a positive match.

On this experiment some changes had to be carried out for the descriptor part of the algorithm. Certain pixels of the model were matched to other pixels of the image provided by the Kinect, even though the object was not in the image. This is because some pixels of the image might have similar characteristics and the algorithm found good matches. The modification then had to be done to consider how many of those matches were in fact good matches. This was accomplished by calculating the amount of descriptors the model had and how many of them had to be close to each other to consider it as a good match.

After all the changes were carried out, the algorithm was able to detect the objects accurately. In the case of the objects recognized with the contour, they had to be exclusively seen and no objects could be on top of them, as this would modify the shape of the contour and the correlation will not provide good values. This was particularly true for the table, as the distribution of the pixels changed if there was something on top of the table, because the other object was considered part of the contour. On the other hand, the descriptors did not cause problems and even if two or three objects were in front of the Kinect, it was actually able of detecting them, having in mind that the correct side of the object was facing the camera.

Both the models for the contours and the models for the descriptors should meet certain characteristics. In the case of the contour, the images should be taken from the extracted contour as it resembles more closely the shape of the object when it would be detected by the Kinect. In the case of descriptors, the images should be also taken with the Kinect but they should be as detailed as possible, due to the fact that having more descriptors, makes it easy to recognize objects.

### 5.4. Performance

As both experiments were applied on a mobile robot, where the position of the objects against the camera changes constantly; the efficiency of the code depends on how the robot moves. In general terms, when the robot is located “properly”; it takes less than 1s to discriminate what a given object is. Now, “properly” is a characteristic that depends on which of the methods is being used to recognize objects.

For contours, properly means that the robot is located in a position where the biggest contour detected is only the object that matches one of the models. In case the extracted contour does not correspond to any of the models, even if the object is present on the image, it will not recognize anything. With descriptors, the position is not that critical but it is important. In the case the robot is not close enough to one of the objects used in this part of the code, then the amount of detected descriptors and its distance would be different from the ones expected. This means that the robot should be within a certain distance from the object, to detect it properly. Furthermore, this distance will depend on the criteria used to detect the object and it is not a fixed value.

At this point it should be considered that the task tries not to deal with false positives, but to make the code in such a way that it would not make mistakes. Since this is applied to a mobile robot, the robot can move in case the distance or the position is not the appropriate one. For example, if the biggest contour extracted does not match any of the models, the robot can move to get a better view of the object or in the case of descriptors, the robot can move closer to an object to find out if it matches one of its models.

Using the results on both experiments, the values for the correlation, descriptor distance and amount of descriptors was narrowed down to values that proved to be effective. In the case of the correlation, the range to detect the objects is in Table 1.

The values used to discriminate objects based on the descriptor part of the code, were also obtained from both experiments. These values are the distance between descriptors and the minimum amount that should be under this distance. Each object has a different amount of descriptors since images do not provide the same results. Table 2 shows these values.

All the values, for contours and descriptors, where obtained empirically. These means that while the robot was moving in both scenarios, the data obtained was used to discriminate the objects. In the case of contours, the values of the correlation changed when the biggest contour was one of the objects to be detected and when there was a different contour. All the contours detected while moving provided different values and the values considered were only the ones when the object was the correct one. Descriptors distance and descriptors amount were obtained similarly, the robot moved around and when the right object was in front of the camera, these values were considered.

All these values show that as long as the proper images are obtained, an object can be identified with one of the methods proposed. For contours, the shape of an object can help to calculate what an object is and generalize it. Descriptors on the other hand, are more specific but they can discriminate objects in a very accurate way.

### 5.5. Comparative Study with RoboEarth

**Figure 26.**
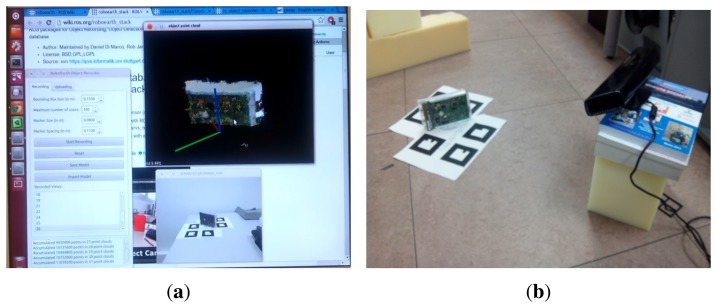
(**a**) Hard drive model; and (**b**) Hard drive modeling.

To check the suitability of the system presented it is compared with the system of objects detection of RoboEarth [27], which offers a similar utility and it is based on ROS architecture, widely used in the world of Robotics. The same objects that were used for the detection test in the previous sections have been modeled. RoboEarth uses the *re_object_recorder* package to create and store object models. This package allows adding to the model point clouds captured with the kinect, up to a maximum of 100 point clouds per object. Figure 26a shows the image of the object generated by accumulating 31 point clouds. The interface does not allow saving a model if there is not enough density of points. To check the effectiveness of this system of object detection, three models were created on the same object as shown in Figure 26b. In one of them the maximum allowed by the interface was reached, 30,720,000 points accumulated in 100 point clouds.

The study obtained specific conclusions on several features:

- Modelling stage. The modelling of objects is a difficult and slow stage. The positioning of the kinect and the tuning up in tilt, distance to object and altitude requires a very strict control. In addition, the system showed tendency to incorporate the surface to the object, as if it was part of it. This made process of modelling even more delicate and tedious.
- Unstable detection. The system proved to have low reliability. One of the experiments placed the camera directly aiming to the object. It is difficult to keep an adequate position for correct detection. Sometimes 5 detections and 46 measurements were obtained and no object was detected. In other experiments, performing a slow sweep with the camera on the object got 35 measurements and only one detected the object.

As conclusion, the system proposed in this article is more stable, faster and with better success rates and at a bigger distance than the alternative system studied. Taking into account the semantic navigation system which is going to be used, the detection of an object should be accomplished simultaneously to the displacement of the robot. With this purpose a fast, stable, and not so dependable on a specific still position to detect objects is more convenient.

## 6. Conclusions

Object recognition is a very difficult task, mostly because the images taken by a camera differ from each other even if taken under the same conditions. Based on this, different approaches have been considered, depending on the type of application where a given project would be carried out. As mobile robots are the scope of this paper, the goal is to provide a fast method to detect objects, so the robot can move faster and this is where less computationally demanding algorithms are needed. Since no interaction with those objects is required, algorithms that provide less amount of data can be used to enhance the speed of the robots movement and at the same time, give accurate results.

The Kinect's depth image, provide just the amount of detail needed to detect objects by contours. SURF was selected as it provides a way to detect all the objects that cannot be generalized using correlation. This is very useful because there are a lot of objects with similar contours that simply with this information, would be very difficult to discriminate. The major advantage about using this method is the fact that changes in the environment should not cause major problems as the algorithm was created to overcome this type of scenarios. What was very useful about both methods is the fact that they are not very computationally demanding, which was one of the constraints of this work. This allows the robot to detect objects in a fairly efficient way and provide results fast enough so the robot can move at a normal pace.

## Figures and Tables

**Table 1.** Range to detect objects. Correlation values.

**Object**	**Lower Correlation Value**	**Higher Correlation Value**
Chair 1	0.9 × 10^9^	1 × 10^9^
Chair 2	0.7 × 10^9^	0.8 × 10^9^
Table	2.2 × 10^9^	2.4 × 10^9^

**Table 2.** Values used to discriminate objects based on descriptors.

**Object**	**Distance**	**Descriptors**
Bottle	0.2	>15
Box	0.17	>2
HDD	0.26	>4
Keyboard	0.2	>73
Screwdrivers	0.24	>2
Source	0.25	>4
Thermal fan	0.18	>130
